# A Systematic Review of the Barriers to the Implementation of Artificial Intelligence in Healthcare

**DOI:** 10.7759/cureus.46454

**Published:** 2023-10-04

**Authors:** Molla Imaduddin Ahmed, Brendan Spooner, John Isherwood, Mark Lane, Emma Orrock, Ashley Dennison

**Affiliations:** 1 Paediatric Respiratory Medicine, University Hospitals of Leicester NHS Trust, Leicester, GBR; 2 Intensive Care and Anaesthesia, University Hospitals Coventry and Warwickshire NHS Trust, Coventry, GBR; 3 Hepatobiliary and Pancreatic Surgery, University Hospitals of Leicester NHS Trust, Leicester, GBR; 4 Ophthalmology, Birmingham and Midland Eye Centre, Birmingham, GBR; 5 Head of Clinical Senates, East and West Midlands Clinical Senate, Leicester, GBR

**Keywords:** ai & robotics in healthcare, health care delivery, literature review, barriers to implementation, machine learning (ml), artificial intelligence in healthcare

## Abstract

Artificial intelligence (AI) is expected to improve healthcare outcomes by facilitating early diagnosis, reducing the medical administrative burden, aiding drug development, personalising medical and oncological management, monitoring healthcare parameters on an individual basis, and allowing clinicians to spend more time with their patients. In the post-pandemic world where there is a drive for efficient delivery of healthcare and manage long waiting times for patients to access care, AI has an important role in supporting clinicians and healthcare systems to streamline the care pathways and provide timely and high-quality care for the patients. Despite AI technologies being used in healthcare for some decades, and all the theoretical potential of AI, the uptake in healthcare has been uneven and slower than anticipated and there remain a number of barriers, both overt and covert, which have limited its incorporation. This literature review highlighted barriers in six key areas: ethical, technological, liability and regulatory, workforce, social, and patient safety barriers. Defining and understanding the barriers preventing the acceptance and implementation of AI in the setting of healthcare will enable clinical staff and healthcare leaders to overcome the identified hurdles and incorporate AI technologies for the benefit of patients and clinical staff.

## Introduction and background

Medicine has embraced technology over the last five decades and many of the most exciting developments are a consequence of this willingness to incorporate new and novel advances into clinical practice. Of the present areas of development and research, artificial intelligence (AI) has garnered the most attention, captured people’s imaginations, and is unique in that it is the only technology that has the potential to revolutionise every area and speciality.

The Dartmouth workshop on “Summer Research Project of Artificial Intelligence” in 1956 is widely considered to be the founding event in the field of AI where this terminology was started [1]. The term AI now refers to computer systems and technologies that behave in ways that resemble processes associated with human intelligence [2]. AI in its myriad guises is already embedded in the financial sector, commerce, and science and is increasingly being employed and investigated in the healthcare sector.

Although AI encompasses a number of technologies. In this literature review, AI mainly refers to machine learning. Machine learning encompasses the use of data and algorithms to mimic the human decision-making process, with the overall aim of improving its accuracy [3] and is one of the most common forms of AI being used in different areas of healthcare. This is being used to predict the success rates of treatment protocols in specific patient cohorts, to predict the risk of disease acquisition using neural network learning, and to predict outcomes by deep learning where machine learning involves multiple variables and features [4].

Unfortunately, new technologies take time to be embedded in healthcare and despite all the theoretical potential of AI, the uptake in healthcare has been uneven and slower than anticipated and there remain a number of barriers, both overt and covert, which have limited its incorporation. The aim of this review is to identify and examine these barriers to help produce a framework for health systems to implement AI.

## Review

Materials and methods

A review of reports published by healthcare organisations, arm’s length health bodies, and think tanks in the UK was undertaken. This helped to inform the search criteria and ensured that the work produced by the East and West Midlands Clinical Senates would add value, avoid duplication of previous reports, and aid the development of the data extraction tool. Organisations included the UK Government and Parliament, NHS England, NHSX, National Institute for Clinical Excellence (NICE), Health Education England, Care Quality Commission (CQC), Academy of Medical Royal Colleges, Royal College of Physicians, Royal College of General Practitioners, National Institute for Health Research (NIHR), Academic Health Science Networks - England, Reform, the Public Service Think Tank, and The Health Foundation. Although this list of healthcare organisations is not exhaustive, it has assisted in providing a greater understanding of the emerging AI healthcare landscape.

Searches

Searches of the following databases were performed on 22/12/2021: AMED, BNI, CINAH, EMBASE, EMCARE, HMIC, MEDLINE, PsycINFO, and PUBMED. The search had no limitations for year of publication, type of study, or language.

The following terms were used:

“Artificial intelligence” OR “deep learning” OR “machine learning”

AND

“Barriers” OR “obstacles” OR “difficulties”

AND

“Implementation” OR “application” OR “utilisation”

Articles Screened by Title and Abstract

Titles and abstracts were reviewed for potential inclusion in the full-text review according to the following criteria: articles highlighting the possible barriers to the implementation of AI in the healthcare setting and all research methodologies were accepted.

Full-Text Article Assessment

The selected full texts were included for further analysis and data extraction was done according to the following criteria: articles that have investigated barriers to the implementation of AI in healthcare, where a full-text version has not been published, an abstract only may only be included if there is a significant discussion about barriers to implementation, and an English language version of the study is available.

This process was done independently by two screeners. Each screener was blinded to the other screener’s decisions and when both screeners had compiled a list of included articles these were compared. Any disagreements between the screening reviewers were resolved by discussion and if necessary, a third reviewer was involved as an impartial mediator.

Secondary Searches

All the references from the articles in the primary search were reviewed. 

Data Extraction Tool

After review of the healthcare organisation reports, each reviewer independently compiled a list of the barriers to the implementation of AI. These were collated and refined into key broad themes of barriers and further divided into sub-themes to produce the data extraction tool that allowed qualitative assessment of the review articles. This framework was used to analyse the articles found during the literature searches.

Data Synthesis Strategy

Data are presented in key themes. We performed a narrative synthesis of the evidence for each of the key barrier themes. 

Results

Search Results

**Figure 1:**
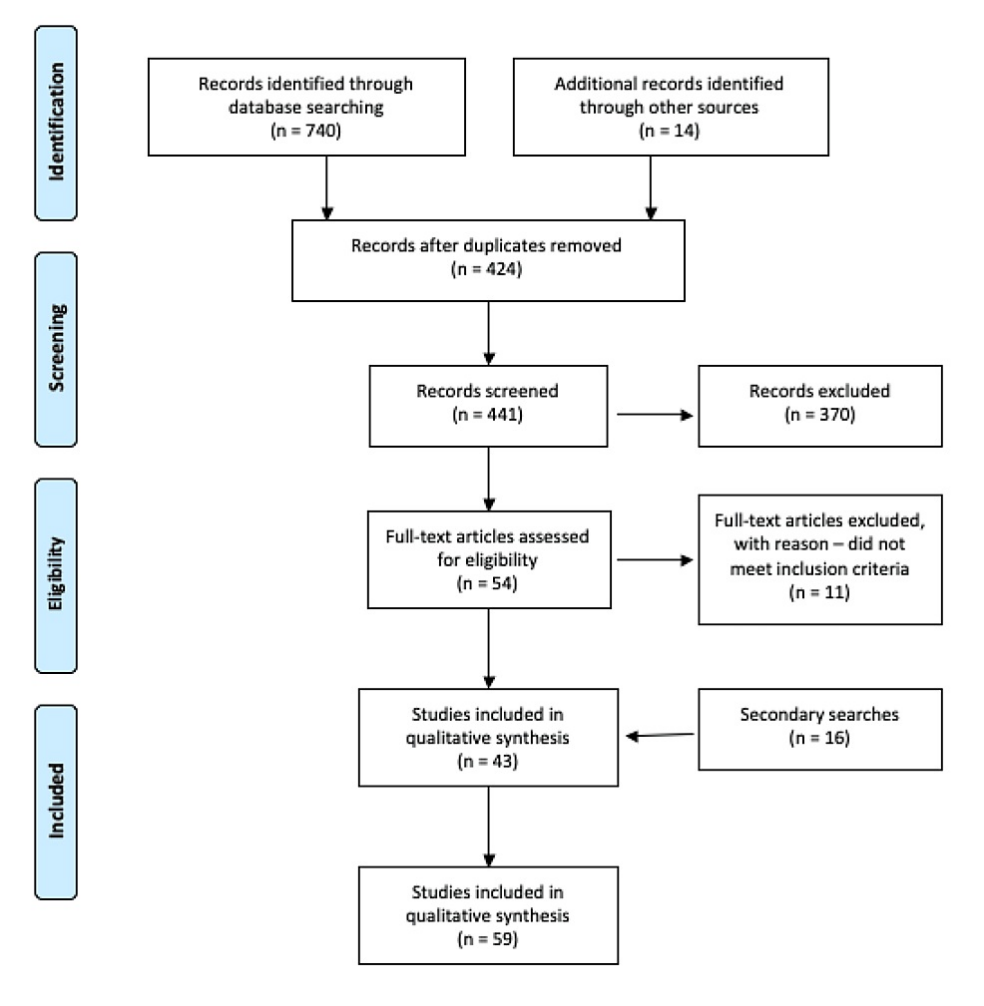
PRISMA flow diagram of article identification, exclusion, and number included for qualitative synthesis PRISMA: Preferred Reporting Items for Systematic Reviews and Meta-Analyses

A total of 43 articles were included for data extraction following a review of the records identified through database searching. A further 16 articles were included following secondary searches; therefore, there were a total of 59 articles included for data extraction. Figure 1 shows the Preferred Reporting Items for Systematic Reviews and Meta-Analyses (PRISMA) flow diagram.

Details of the articles and the barriers noted in these articles are highlighted in Table 1.

**Table 1:** List of reviewed published papers and thematic barriers

Author	Year	Journal	Thematic barriers					
			Ethical	Technological	Liability and regulatory	Workforce	Social	Patient safety
Abràmoff et al. [5]	2020	American Journal of Ophthalmology	x	x		x		
Ahmad et al. [6]	2020	Techniques in Gastrointestinal Endoscopy	x	x	x	x	x	x
Ahn et al. [7]	2020	Hepatology		x	x	x	x	
Alexander et al. [8]	2019	Journal of the American College of Radiology	x	x	x	x		
Angehrn et al. [9]	2020	Frontiers in Pharmacology	x	x	x	x	x	
Arora [10]	2020	Medical devices: Evidence and Research	x	x				x
Baxter et al. [11]	2020	ACI OPEN		x		x		
Ben-Israel et al. [12]	2019	Artificial Intelligence in Medicine	x	x	x			
Brady et al. [13]	2020	Diagnostics	x	x	x	x	x	x
Bzdok et al. [14]	2017	Biological Psychiatry	x	x			x	x
Challen et al. [15]	2018	BMJ Quality and Safety		x	x	x		x
Chan et al. [16]	2019	British Journal of Radiology		x	x			
Char et al. [17]	2018	New England Journal of Medicine		x	x	x		
Chen et al. [18]	2020	Journal of Medical Internet Research	x	x	x			x
Chu et al. [19]	2020	Journal of Ophthalmology					x	
Draffan et al. [20]	2019	Technology and Disability	x				x	
Faes et al. [21]	2019	Lancet Digital Health	x	x	x	x	x	
Gearhart et al. [22]	2020	Cardiology in the Young	x	x	x	x		x
Giannini et al. [23]	2019	Critical Care Medicine		x				
Gomolin et al. [24]	2020	Frontiers in Medicine	x	x	x		x	
Grant et al. [25]	2019	Annals of Emergency Medicine	x	x	x			x
Greenes et al. [26]	2001	Studies in Health Technology and Informatics		x	x			
He et al. [27]	2019	Nature Medicine	x	x	x	x	x	x
Ho et al. [28]	2019	Clinical Radiology	x	x	x	x		
Kelly et al. [29]	2019	BMC Medicine	x	x	x	x	x	x
Kras et al. [30]	2020	AI in Retina	x	x	x			x
Lai et al. [31]	2020	Journal of Translational Medicine	x	x	x			x
Liberati et al. [32]	2017	Implementation science	x	x	x	x		x
Linthicum et al. [33]	2018	Behavioural Science and Law		x	x	x		
Loh et al. [34]	2017	The Journal of mHealth				x		
Loncaric et al. [35]	2020	Revista Española de Cardiología	x	x		x	x	x
Longoni et al. [36]	2019	Journal of Consumer Research	x	x				
Marcu et al. [37]	2019	Health and Technology	x	x	x			
McCradden et al. [38]	2020	Lancet Digital Health	x	x				x
Miotto et al. [39]	2017	Briefings in Bioinformatics	x	x				
Nemanti et al. [40]	2018	Critical Care Medicine		x			x	
Panch et al. [41]	2019	Digital Medicine	x	x	x	x		x
Paranjape et al. [42]	2020	American Journal of Clinical Pathology		x		x		x
Pesapane [43]	2018	Insights into Imaging	x	x	x		x	
Petitgand et al. [44]	2020	Digital Personalized Health and Medicine	x	x	x	x	x	x
Prosperi et al. [45]	2018	BMC Medical Informatics and Decision Making	x	x	x			
Ryan [46]	2020	Science and Engineering Ethics	x	x				
Sakamoto et al. [47]	2020	Translational Lung Cancer Research	x	x	x	x		
Scarpazza et al. [48]	2020	Translational Psychiatry		x		x	x	x
Sendak et al. [49]	2020	JMIR Medical Informatics	x	x		x		
Sendak et al. [50]	2020	The Journal for Electronic Health Data and Records		x		x	x	
Shalaby et al. [51]	2019	Clinical Radiology		x	x	x		
Shimizu et al. [52]	2020	Cancer Science	x	x		x		
Singh et al. [53]	2020	Translational Vision Science and Technology	x	x	x	x		
Stead [54]	2018	Journal of American Medical Association			x	x		
Strohm et al. [55]	2020	European Radiology	x	x	x	x		x
Tang et al. [56]	2018	Canadian Association of Radiologists Journal	x	x	x	x		x
Thompson et al. [57]	2020	Bulletin of World Health Organisation	x	x	x			x
van Assen et al. [58]	2020	Journal of Thoracic Imaging	x	x	x	x		x
Varghese [59]	2020	Visceral medicine		x				
Wangmo et al. [60]	2020	BMC Medical ethics	x	x	x	x		
Waring et al. [61]	2020	AI in Medicine		x		x		x
Willemink et al. [62]	2020	Radiology		x				
Zeng-Treitler et al. [63]	2019	Journal of Medical Internet Research	x	x	x	x	x	

Themes

Six key themes were identified from the review of background literature that formed a framework which was used to analyse the articles. There were several key themes (and a number of articles discussing the theme) as follows: ethical barriers (39 articles), technological barriers (55 articles), liability and regulatory barriers (37 articles), workforce barriers (35 articles), patient safety barriers (24 articles), and social barriers (18 articles). These themes are explored in the discussion.

Discussion

The key themes identified from the literature are discussed in the order of prevalence within the literature. There was overlap between the themes and the key aspects are explored further.

Ethical Barriers

There are significant ethical concerns that act as barriers to the implementation of AI in healthcare and 39 studies reviewed referred to these ethical barriers. Within the theme, the most prominent were privacy, trust, consent, and conflicts of interest.

Twenty of the studies in this review have highlighted that privacy may be a significant barrier when implementing AI in healthcare. The creation of accurate AI algorithms requires that developers have access to large datasets for training and there is an understandable apprehension that the use of this data may sometimes be at odds with the patient's right to confidentiality [25]. In the European Union, the General Data Protection Regulation (GDPR) is clear and states that “patients own and control their own data and must give explicit consent for its use or when it is shared [64]”.

It is essential that patients are fully informed about the fate of their data, and it must be mandatory that they consent for its use when it is to be shared with AI developers. They should understand who can access their data, how it will be stored or used, and how their privacy will be protected [13,29]. 

Data ownership and consent to store data was highlighted as a concern in 10 studies. He et al. suggested that with wider sharing of data, there is a need to redefine the concepts of patient confidentiality and data privacy [27]. Discussions concerning the development of cybersecurity measures are inevitably going to become increasingly prominent to address emerging issues of improper data sharing [27].

There is a need for clear governance of AI implementation in healthcare that clarifies the role of all stakeholders in data ownership and use [65]. Marcu et al. highlighted the importance of having data privacy policies for secure data storage, usage, and sharing to avoid breaches of sensitive confidential data [37].

Another barrier noted in 25 of the studies was concerns about whether AI technology could be “trusted”. Chen et al. noted that doctors struggled to trust AI for several reasons [18]. Firstly, health professionals only have limited or no training in evaluating AI software. Most of the evidence relating to the use of AI in healthcare is retrospective and there is a paucity of randomised controlled trials on AI for clinicians to appraise. If these technologies do not have a rigorous evaluation prior to their introduction and use in the real world, this could lead to significant patient harm, irreversible loss of confidence for the medical profession, and inaccurate conclusions being made at the population level [6,18]. There are also concerns that as these technologies are often being developed commercially with the main driver being profit for the parent company, this may affect the level of scrutiny that they undergo prior to their release into the clinical environment [27,66].

Secondly, the overwhelming majority of algorithms have a lack of interpretability or transparency, and developers cannot (or will not) explain the basis of their decision making, the so-called black box phenomenon [27]. Sakamoto et al. suggested that this could lead to issues with both validation and clinical oversight [47]. It may also affect the doctor-patient relationship as clinicians may be unable to explain decisions made by AI algorithms to their patients [31].

Another potential problem is the utility of AI in complex multisystem diseases. Health professionals may be reluctant to adopt AI into practice if they feel that the system is not sufficiently flexible or sophisticated to cope with complex conditions especially if the decisions cannot easily be reviewed [36] and under these circumstances, trust becomes a significant barrier.

Conflicts of interest have been highlighted as a barrier to AI implementation in five studies. Brady et al. highlighted this as a current issue for radiologists who may have commercial conflicts of interests by implementing AI; they may be involved in developing or potentially may even receive royalties from. They suggest that this issue should be addressed in an analogous fashion to issues of conflicts of interest while developing medications or medical devices [13].

There is no overall consensus on how to deal with these complex ethical dilemmas, particularly in the absence of a strong regulatory framework and the lack of agreement among experts. It is however increasingly being recognised by AI developers that ethical analysis is difficult to incorporate into AI development. A new concept of algorithmic fairness is being incorporated where the AI technologies are focussed on identifying any latent biases and demonstrating an absence of between-group bias [38]. However, this approach is not infallible and ethical dilemmas are inevitably going to emerge while implementing AI technologies in real-world healthcare settings in different patient populations. These ethical dilemmas particularly surrounding privacy, trust, transparency, and accountability are major barriers to the implementation of AI in healthcare settings. In the future, they will have to be addressed at the stage of AI development and incorporated into the training of healthcare professionals.

Technological Barriers

Technology is a central and prominent theme of barriers to AI. Forty-two articles discussed data barriers including data quality, data access, and data-set size. Twenty-three articles examined how AI’s application can be a barrier with issues relating to interoperability, usability, and integration into current workflows. Validation of data was raised by 18 articles and 15 of those discussed hardware and infrastructure. There were other barrier themes that emerged and are discussed in this section which are also explored elsewhere including explainability and transparency.

Data quality, accuracy, and dataset size: Implementing large data sets into AI represents a considerable challenge. It is difficult to acquire and amalgamate large, high-quality data sets that are complete and diverse [12,18,47]. AI platforms are limited by the concept of “what goes in is what comes out” implying that the algorithm is only as good as the data source “teaching” it [53].

Apart from data quality, data quantity is relevant for the training of machine learning algorithms. When small data sets are used to validate AI there are inevitable problems. AI may not be able to differentiate between variations of normal and abnormal, it may not be able to allow for the effect of known confounders such as gender, age and ethnicity [35,48,53] or unknown confounders which will reduce generalisability [29]. It may also be blind to biases from using retrospective data, underrepresented patients, or medication selection. 

Many conditions or diagnoses cannot be clearly defined, leading to imprecise phenotyping of diseases and their treatments [45]. There is also substantial variability between experts who evaluate data, and this variability may result in biased labels. This cannot be completely avoided but can be mitigated by having multiple experts evaluating the same case. Additional biases occur including over-fitting which results from the application of AI trained in one population to a different population without retraining on the new dataset [49]. A balance needs to be found between widening access to data while ensuring confidentiality and respect for privacy. Recurring concerns are also raised regarding the utilisation of non-standardised reporting standards (or those that are not pre-defined) within datasets and with different sample characteristics [30].

These arguments expose the brittleness of AI, where the tool which has been developed only works on the training data and is easily misled. Even with large datasets, AI is not infallible as the high-profile failure of GoogleFlu demonstrated [18].

Although a huge number of guidelines do exist, often in narrative form, there remains a paucity of coherent consensus on the most effective of these guidelines and applications [26]. Willemink et al. suggest that nearly all limitations can be attributed to one substantial problem, the lack of available data for the training and testing of AI algorithms [62]. There is a clear lack of variety in the meta data and quality of information and in addition there is continued use of outdated machines, low-quality data, and the use of numerous open-source data sets restricted to research only use.

Future aims should include the creation of data systems that will channel prospective data into AI algorithms that will prospectively adapt and change. To aid future comparisons, AI systems should be tested on analogous datasets so that performance between systems can be assessed and compared. Ultimately, there is a need for high-quality data to be standardised.

The data AI is trained on is important. The quality and quantity of data on which the algorithms are trained are two important determinants of the performance and generalisability of the algorithm. Future AI datasets will need to include aggregates of multiple data sets that ensures algorithms match the depth and breadth of a doctor’s knowledge.

Interoperability and usability of data and its integration into the workflow: A clear example of an interoperability barrier is seen where AI is applied to medical imaging. A major limitation of current commercial imaging systems is that they acquire annotations and storing them in a format which does not permit reuse in AI development [62]. The lack of data interoperability between systems and the lack of standardisation in entered data ultimately affects the quality of predictive AI models [63]. These models are yet to be integrated into clinical workflows in a fashion that improves clinical care or outcomes [44] and there remains a divide between those using and those implementing the AI models providing a significant challenge for those responsible for the model when it is used clinically.

Data validation: Validation of AI efficacy following its development is also a barrier and there is a lack of high-quality evidence and data to support AI systems use, with limited randomised controlled trials and a lack of prospective data with virtually all data to date having been acquired retrospectively [25]. Ultimately, well designed, multi-site, multi-centre (ideally heterogenous population) validation studies will be necessary to understand the real-world impacts of AI in healthcare and explore robustness, interpretability, and trust of data.

Hardware and infrastructure: There is currently no clear commercial or institutional leader in the field of healthcare AI. This lack of established leaders of AI in individual countries and national healthcare systems results in the use of different products to address single areas of need, making the identification, selection, and utilisation of AI extremely challenging.

Presently, no AI programme continually receives feedback about its diagnostic performance, a task that is essential to learning from experience. In addition, a major technical challenge is the development of “a reasonable” detection rate of abnormalities without an excessive rate of false-positive findings compared with a comparable clinician-based system.

The widespread implementation of AI will require substantial reconfiguration of IT systems which will have resource implications and currently it is difficult if not impossible to allocate sufficient investment where there are competing demands for healthcare equipment. Moving forward there are vital prerequisites including support systems, strategic plans, and budget planning [42] and the infrastructure for data sharing and secure transfer of data is costly and presently lacking [6]. There is also the risk of data breaches which reduces the number of organisations who will be prepared or able to adopt even the most promising initiatives.

Three papers discussed security being an issue relating to AI implementation. As AI becomes increasingly widespread, algorithms may be the target of cyber-attacks and there is concern that AI models could subsequently cause deliberate harm or be used for fraudulent purposes [6]. A particular challenge that emerged from the review of the literature is the issue organisations face when trying to assess myriad vendors marketing AI platforms in the absence of any established databases or source of information and feedback relating to AI vendors and companies [53].

Explainability, transparency, and the black box theory: Another barrier to AI implementation was explainability, transparency, and the complex issue of the black box theory. The “black box” phenomenon refers to when an AI algorithm reaches a conclusion without users being able to understand the mechanism or basis or ‘see inside’ the system [67]. This concern in explaining AI decision making exposes the technical vulnerabilities with a lack of human-interpretable explanation for model predictions and is strong evidence that significant issues remain before AI is in apposition to replace clinicians [51]. There also remain concerns about the discordance between scientific advances and the on-going announcements made in the media about advances in AI and there a persistent tendency to focus on the innovative aspect of the tool and not its utility [31].

Liability and Regulatory Barriers

The theme of regulation was prominent with 37 papers discussing this issue and describing it as significant. Within the theme, the most important sub-theme (30 papers) was the question of who or what was responsible for the outcomes and decisions provided by an AI. The other prominent sub-theme was regulation and legal aspects of AI, which includes data governance (23 papers).

Liability: Addressing liability relating to the use of AI is a particular challenge and clinical staff are used to taking responsibility for their decision making [13]. The issue becomes more complex when decision-making is guided by, or even entirely devolved to AI and questions relating to who or what is liable for a bad outcome where AI have been utilised are ubiquitous. Candidates include the clinicians who used the AI tool, the software developer who designed and developed the system, the vendor who retails the product, the healthcare service that purchased it or the regulator who approved it [28,33]. This issue remains unanswered due to the combination of the legal and ethical questions raised and presently healthcare professionals remain liable for the decisions they make, even if based on an AI algorithm for which they may have little or no insight.

This lack of attributable accountability is likely to prevent those medical professionals who are currently responsible for clinical decisions from embracing the technology. Contrary to this issue however, if a professional fails to use or abide by the advice of an AI tool and there is a poor outcome, it is conceivable that this may be considered clinical negligence [68]. Consequently, the serious complications that may occur as a result of these decisions will deter physicians who will inevitably not be prepared to be responsible for outcomes from the use of new and emerging technologies which function and are tested in ways they do not fully understand and are not privy to.

Regulation and legal barriers: For users and developers, both regulation and a lack of regulation can act as barriers to the adoption of AI in healthcare. Systems and professional regulators need to properly understand and trust the operation of the AIs that will be employed in their speciality to be able to regulate effectively. Unfortunately, transparency is lacking or often completely absent in most of the presently available technologies [12,31]. This is another consequence of the “black box” problem where the inner workings of a system are not understood and therefore not trusted. This is a prominent barrier and has a profound impact influencing whether society and regulatory bodies are prepared or able to trust an AI in routine clinical practice.

The absence of a specific approval system for AI is largely based on this lack of trust, but even where they exist the delay of approval can hinder progress often for considerable periods [47]. There are additional challenges for regulators due to the lack of an agreed framework for the evaluation of the performance of these algorithms with no “gold standard” or accepted baseline for comparison [12,30]. Further questions remain regarding a method to continuously regulate AI algorithms during cycles of iterative modifications and updates.

Regulation aimed at protecting commissioners and patients results in barriers for developers and it is accepted that rigorous top-down regulation could stifle the AI innovation. In 2021, the UK Government published guidance outlining their expectations for AI development in the NHS, covering the need for the transparency of algorithms and accountability [69]. The onus is on developers to meet these regulatory requirements, although the guidance acknowledges that it can become complex when a product performs actions that fall under the aegis of multiple regulatory bodies.

Data and information governance is also perceived as a significant barrier to the implementation of AI systems. The GDPR guidance outlines a comprehensive set of regulations for the governance of personal information which became effective in May 2018 [64] and influences AI implementation in several different ways [21,27]. Specific arrangements may therefore need to be developed to provide necessary protection for patients, organisations, and clinicians using data for AI systems [17,41]. The most appropriate regulatory approach for AI remains to be clarified but will need to be addressed to remove this hurdle and encourage developers [25].

Workforce Barriers

The theme of workforce is considered an important barrier to the adoption of AI with 35 studies citing it as a major concern. Within the theme, the education and training of the workforce was the most prominent aspect referred to in 27 studies. Other barriers identified related to workforce and there was considerable overlap; willingness/engagement of the workforce (13 studies), fears of job displacement (9 studies), finances (7 studies), and time (6 studies).

Education and training: The education and training of the healthcare workforce is a significant barrier to the adoption of AI and was highlighted in 27 studies. Detailed knowledge regarding the potential and workings of AI in the medical community remains rudimentary and considerable AI education and training will be needed [42]. The varying levels of technological literacy, satisfaction with and understanding of the existing technology will need to be overcome before the workforce that will be required to use the technology are comfortable or competent in clinical practice [41,53]. Unfortunately, frustrations may potentially become more acute as physicians learn how to incorporate AI platforms while still struggling with existing healthcare technologies [53]. Healthcare providers will need to develop training programmes specifically targeted at clinicians required to use AI systems and designed to ameliorate the multiple concerns resulting from unfamiliar technology [33,48].

Clinical staff will also need to be educated to enable them to critically analyse the opportunities, pitfalls, and challenges associated with AI [6,28]. Unfortunately, today’s medical education system is lacking in AI training representing a significant barrier in both the medium and long-term. Future medical undergraduate and postgraduate curricula should be updated to include a basic understanding of AI methodology and limitations [6,53,56] including advanced statistical and computational skills. Some medical schools have already begun to include AI education courses in their curricula, although educators do not fully understand how AI will affect clinicians’ roles. In addition, there are limited individuals in medical faculties who are AI competent and capable of teaching the relevance and importance of AI in the healthcare setting [53]. The barrier resulting from a lack of education also extends beyond clinical staff, as specific technical expertise and mathematical knowledge is required to develop and utilise AI tools and proficiency is still rare within healthcare settings [21].

Willingness/engagement: Physician ‘buy-in’ and engagement remains a necessary and uniquely challenging barrier for AI and was discussed in 13 papers. Some physicians do not engage fully with a new AI process [11] and this lack of engagement may produce clinical problems and ignorance of or reluctance to understand AI could lead to poor patient outcomes. Without ensuring adequate engagement of clinical staff and healthcare leaders even the best AI tools are unlikely to be accepted and integrated and will be unable to improve clinical outcomes.

When a system is perceived by clinicians as threatening and interfering with autonomy and critical thinking, uptake will inevitably be compromised and consistent and determined effort will be essential to nurture clinicians’ engagement with the AI tool [32]. An additional but separate issue for a number of clinical staff is scepticism regarding the current capabilities for AI in particularly complex patients or with conditions where management is known to be difficult requiring input from multiple specialities to achieve the best outcome.

Engagement of clinicians can also be affected by the required algorithm-human interaction with the AI technology. Information overload or alert fatigue are barriers to using AI tools [53,63]. Further work is required to understand this algorithm-human interaction to ensure maximum benefit is derived from the technology and system development should consider the need to produce systems which are user-centred.

Job displacement: The fear of job displacement is a consistent theme and clinicians are not immune to the fear of unemployment or radically changed job plans arising from the emergence of increasingly complex and competent AI. This concern could theoretically lead to behaviours and practices in the future designed to ensure the continuing relevance and roles of human practitioners in healthcare [13].

In clinical specialities where investigations and results can be readily digitised and interpreted by autonomous AI there are already concerns and ophthalmologists, cardiologists, pathologists and radiologists [13,35] fear deskilling and the change in their role in clinical pathways. As technologies improve this could lead to over-reliance on non-clinical systems [6,35] and AI needs to be used appropriately and sensitively, allowing the role of healthcare providers to evolve with their introduction with the new technology augmenting rather than replacing clinical staff. Whether AI systems will eventually replace radiologists is the wrong question, the more apt question to be asked is “will radiologists who use AI replace radiologists who don’t” [70].

Finances: Workforce funding limitations are a clear barrier even when projected efficiencies from the introduction of AI systems are considered. Expert input for training data for AI algorithms is very expensive to obtain (58 studies) and AI maintenance activity will require significant ongoing funding [27,49]. Health system leaders must prepare for significant investment in personnel and technology to test, validate, implement, and improve AI tools. Calculating the impact of the changes and allocating healthcare budgets appropriately will be confounded by the likelihood that the benefits and costs of using AI may be unequally divided between departments and providers [69].

Time: The time required for the training of AI systems and certainly in the early stages following implementation needed to utilise the systems is likely to remain a barrier for the foreseeable future. Initially AI will require data, some of which will have to be collected by the healthcare workforce. In one study of the implementation of a decision support AI tool, the largest resource barrier was found to be personnel time [49] and tasks such as labelling of raw data are tedious and time-consuming and will not encourage staff to embrace the new technology [27,63]. The education and training of the healthcare workforce has been discussed in the context of the expertise not being presently available, and the topic not included in curricula, but it will also be important for healthcare leaders and clinicians to engage with developers and providers to ensure that adequate funding and time is allocated.

Patient Safety Barriers

It is believed that AI will have a positive impact on improving patient safety with several studies demonstrating that its utilisation will reduce mortality and improve diagnostic speed and accuracy [71]. AI however is not a panacea and 24 studies raised concerns about the impact of AI on patient safety and these safety issues will need to be carefully considered during introduction into clinical practice [15]. Failure to mitigate these safety issues could dramatically affect the implementation of the technology in healthcare with patients and staff rapidly losing confidence.

Distributional shift: The first patient safety issue to highlight is the concept of “distributional shift” which may be familiar to clinicians who find that their previous clinical experience may be inadequate when encountering new situations [15]. Clinicians are used to working in these difficult environments with limited or poor data and are good at weighing up the cost and consequences of their decisions as well as the impact of false positive or negative results on their patients’ condition. This can often lead to a cautious approach, especially when there is uncertainty in the diagnosis or the best management whereas contrasted with Al algorithms which are best utilised on controlled, curated datasets similar to the data with which they were trained. Real-world data is also likely to be less tightly grouped and will display regional and population variations and be influenced by the equipment being utilised and differing levels of curation. AI systems do not reliably recognise important context or a pertinent change in data that should alter its predictions. Secondly, most tools perform a region of interest analysis to investigate a single disorder of interest and are not created to investigating multiple disorders or complex multisystem complaints synchronously. This can result in a system that fails to identify secondary pathologies or that will confidently make incorrect predictions without considering individual context, misleading clinicians and acting as a barrier with clinicians reluctant to consider its use in the future.

Automation bias: As AI becomes increasingly accepted and incorporated into healthcare management, there is a risk that clinicians will become complacent and accept that guidance from an automated systems is infallible and consequently fail to search for confirmatory evidence, a practice known as automation bias [18,47]. This situation may deteriorate over time as algorithms age and may cease to recognise changes in diseases, clinical practice and medication and may reinforce outdated or inappropriate practice. For example, when a usually reliable AI system encounters unrecognised data, it may not ‘fail safely’, but make erroneous predictions which may not be questioned by the busy clinician who would previously have considered alternative diagnoses or treatments. Another concern is that established AI algorithms may be poorly receptive to new medical innovations as there would be no prior data to retrain the system inhibiting medical innovation and preventing it being safely introduced into daily practice.

Improvements and evolution of AI algorithms: AI algorithms will use whatever signals are available to achieve the best possible performance, which is why they have been so well utilised playing chess, poker, and alpha go. They can dynamically learn new rules and explore new techniques to improve their performance, the key to mastering games, but this may be a significant risk in healthcare [72]. AI in healthcare may attempt to ‘game the system’ and learn to deliver results that appear successful in the short-term fulfilling their initial remit but running contrary to longer term health goals or stopping the system becoming generalisable. For instance, in one classic example, an AI model did not learn the intrinsic difference between dogs and wolves, but instead learned that wolves are usually pictured standing on snow, while dogs usually appear on grass [29]. Further concerns have also been raised on the ability of autonomous AI systems to safely push the boundaries of treatments to discover new strategies to improve their current outcomes. Although this may lead to eventual improvements in care there is also a risk it will lead to multiple failures and there must be strict guidelines detailing which new strategies are safe to explore.

It is beyond doubt that AI technologies have the potential to irreversibly change medicine and bring about improvements in patient management and diagnosis. It is nevertheless important that AI systems are designed to constantly assess their own confidence in the predictions they make and to include failsafe behaviours in which they would refuse to make a diagnosis if there was poor data or low confidence in the end result. This section also indicates the need for truly “explainable AI”, rather than the opaque black box algorithms that do not allow for interrogation of the decisions that have been made. Understanding the algorithms would allow humans to assess the likelihood of a level of confidence for each diagnosis in real-time rather than when a mistake has been made. It is crucial that patient safety remains at the heart of AI both for the management of individual patients and because any safety issues, particularly any which are high profile or enter the public domain would have a dramatic and immediate effect on confidence in the technology and could act a barrier to the technology's eventual success. This is analogous to a self-driving car which even if orders of magnitude safer than a human driver would instantly become difficult to implement if it resulted in the death of a pedestrian.

Social Barriers

The conditions to which we are born, live, and work effect all aspects of our mental and physical wellbeing and our ability to access equitable healthcare. Health inequalities are unjust and avoidable differences in health across the population and between different groups within our society and are unacceptable.

This gap in health can be further exacerbated by healthcare bias, where patients receive different standards of care because of their race, age, gender, or other characteristics. It is therefore of paramount importance that any new technology does not contribute to the inequalities in society and actually aims to reduce the inherent bias in a healthcare system. Health inequalities and healthcare bias was discussed in 19 articles in the context of acting as a barrier to the successful implementation of AI in healthcare.

Selection bias: There is huge potential that the under-representation of different cohorts in the initial formation of an AI model will lead to reduced accuracy and efficiency in these groups, a form of selection bias. For example, a machine learning algorithm was found to be less accurate at the detection of melanoma in darker skinned individuals, as it had mainly been trained on fair skinned patients [69]. It is also mandatory that vulnerable and minority groups are engaged by developers of machine learning and artificial intelligence models to ensure the data fully capture the diverse healthcare landscape and avoids perpetuating healthcare bias. As a minimum full disclosure of the demographics of patients that have been used to train and validate these models would go some way to ensuring that they match the target population and reduce inherent selection bias which would render the technology unfit for purpose [19].

McCradden et al. raised some interesting points in this respect and acknowledges that in many cases it is difficult to distinguish between acknowledging difference and propagating discrimination [38]. The difficulty arises when trying to ascertain if these differences may have a causative and tangible effect on the outcome. For example, acknowledging that gender may impact the effect of pharmacological compounds is not unjust and would ensure the safest algorithm was created. However, including non-causative factors in algorithm design would lead to no tangible benefit and could lead to further health-related discrimination. One possible way to help mitigate some of these issues is to ensure AI products use fairness solutions to help create bias-neutral models. These solutions can omit variables such as gender or race from the model, derive outcomes that are independent of these factors after controlling for their estimated risk, or mathematically balance benefit and harm to all groups [38].

Digital health inequality: Individuals may have different access to AI driven healthcare, due to cultural, geographic, economic, or a number of other factors. As Ahmad et al. observed there is a risk that a two-tier health system could emerge where more AI-enabled healthcare organisations provide superior patient care and better outcomes [6]. The NHS should strive to “level up” infrastructure equally across the country to ensure that there is not a postcode lottery in respect of access to these new technologies. There also needs to be a concerted effort to reduce widening digital exclusion that would limit the uptake of these technologies especially in vulnerable groups.

Social acceptance: Ten studies indicated that social acceptance of artificial intelligence models remains a barrier to implementation. Singh et al. states that the aim of AI implementation is to create value for the organisation making healthcare more affordable and efficient by identifying key performance indices and tracking return on investment [53]. However, the ‘black box’ nature of AI acts a significant barrier to its social acceptance, and it is not clear whether patients will be willing to accept a diagnosis from a computer instead of one from a clinician especially if it is perceived that it is designed to save time and money without clear evidence that quality has not been compromised. In an autonomous diagnostic setting, will patients depend on non-expert device operators for comfort and clarification [53]. Loncaric et al. have predicted that there will be a loss of the holistic medical approach as healthcare shifts towards data analytics and away from patient interactions [35]. However, the study notes that presently patient preference is dramatically weighted towards human interaction rather than automation, even if it means lower performance, as above all else as humans unlike presently available machines are able to provide gentleness and compassion [35,36].

## Conclusions

The significant barriers to implementing AI in healthcare have been highlighted and discussed in this review. Defining and understanding the barriers preventing the acceptance and implementation of AI in the setting of healthcare is a crucial first step enabling clinical staff and healthcare managers to overcome the identified hurdles. The development of strategies and systems to overcome these barriers will be the real challenge. Gaining a complete understanding of the barriers to using AI in healthcare is an important consideration for those designing and delivering healthcare within rapidly changing healthcare systems. It is especially important to understand which of the barriers are the most relevant in real-world health settings so strategies can be developed to overcome them.

AI faces significant barriers that need to be overcome before its widespread, safe, and successful use is achieved in healthcare. Its expansion seems inevitable, but only by addressing these barriers thoughtfully can the potential of AI be used for the good of the health of the population and the healthcare system.
